# From Cerebrospinal Fluid Neurochemistry to Clinical Diagnosis of Alzheimer’s Disease in the Era of Anti-Amyloid Treatments. Report of Four Patients

**DOI:** 10.3390/biomedicines9101376

**Published:** 2021-10-02

**Authors:** Ioanna Tsantzali, Fotini Boufidou, Eleni Sideri, Antonis Mavromatos, Myrto G. Papaioannou, Aikaterini Foska, Ioannis Tollos, Sotirios G. Paraskevas, Anastasios Bonakis, Konstantinos I. Voumvourakis, Georgios Tsivgoulis, Elisabeth Kapaki, George P. Paraskevas

**Affiliations:** 1 2nd Department of Neurology, School of Medicine, National and Kapodistrian University of Athens, “Attikon” General University Hospital, 12462 Athens, Greece; docjo1989@gmail.com (I.T.); elenisideri1985@gmail.com (E.S.); amavro01@hotmail.com (A.M.); dkfoska@gmail.com (A.F.); iw_toll@yahoo.gr (I.T.); bonakistasos@med.uoa.gr (A.B.); cvoumvou@otenet.gr (K.I.V.); tsivgoulisgiorg@yahoo.gr (G.T.); 2 Neurochemistry and Biological Markers Unit, 1st Department of Neurology, School of Medicine, National and Kapodistrian University of Athens, “Eginition” Hospital, 11528 Athens, Greece; fboufidou@med.uoa.gr (F.B.); myrtop@yahoo.com (M.G.P.); sotirispar5@gmail.com (S.G.P.); ekapaki@med.uoa.gr (E.K.)

**Keywords:** Alzheimer’s disease, beta amyloid, tau protein, phospho-tau, cerebrospinal fluid, biomarkers, anti-amyloid antibodies, aducanumab

## Abstract

Analysis of classical cerebrospinal fluid biomarkers, especially when incorporated in a classification/diagnostic system such as the AT(N), may offer a significant diagnostic tool allowing correct identification of Alzheimer’s disease during life. We describe four patients with more or less atypical or mixed clinical presentation, in which the classical cerebrospinal fluid biomarkers amyloid peptide with 42 and 40 amino acids (Aβ_42_ and Aβ_40_, respectively), phospho-tau (τ_P-181_) and total tau (τ_Τ_) were measured. Despite the unusual clinical presentation, the biomarker profile was compatible with Alzheimer’s disease in all four patients. The measurement of classical biomarkers in the cerebrospinal fluid may be a useful tool in identifying the biochemical fingerprints of Alzheimer’s disease, especially currently, due to the recent approval of the first disease-modifying treatment, allowing not only typical but also atypical cases to be enrolled in trials of such treatments.

## 1. Introduction

Alzheimer’s disease (AD), the most common cause of dementia, is a neurodegenerative disorder characterized by neuronal and synaptic loss and eventually brain atrophy, due to extracellular polymerization and the accumulation of amyloid peptide with 40 and especially 42 amino acids (Aβ_40_ and Aβ_42,_ respectively) in the form of amyloid plaques and intracellular polymerization of hyper-phosphorylated tau protein in the form of paired helical filaments, viewed microscopically as neurofibrillary tangles [1]. This pathophysiological/pathobiochemical process of AD starts many years before, and likely, one to three decades prior to symptom onset [2,3]. Following this long asymptomatic or “preclinical” phase of the disease [4], the symptomatic phase starts [5] initially with mild cognitive impairment (MCI) [6] and finally dementia [7]. At the symptomatic phase, the typical presentation of AD is usually of the “hippocampal amnestic-type”, characterized by a deficit in episodic memory with difficulty in both free and cued recall [8]. However, in approximately 10–15% of AD patients, atypical (non-amnestic) presentations have been described [5] and this percentage may rise to 22–64% in early-onset (pre-senile) cases [9]. Such atypical presentations include primary progressive aphasia (PPA) [10], frontal dementia which may mimic frontotemporal degeneration [11], corticobasal syndrome (CBS) [12], and posterior cortical atrophy [13]. Furthermore, cases of AD mixed with cerebrovascular disease [14], Lewy body pathology [15], and even normal pressure hydrocephalus (NPH) [16] are not uncommon, especially in the elderly. Thus, AD is no longer viewed as synonymous with amnestic dementia [17]. It may be viewed as a biological process, irrespective of the presence (or absence) and the type and severity of symptoms at a certain time point during disease evolution and progression [18]. Then, how can we diagnose AD?

As in any aspect of medicine, the initial approach is always clinical and, clinical criteria formulated more than 35 years ago [19], may show a diagnostic accuracy > 90% when typical patients are examined in specialized centers [20]. However, in the community, in early disease, in atypical or mixed cases, and the presence of comorbidities, diagnostic accuracy may decrease substantially [21]. Thus, it has been estimated that up to 30% of patients with a clinical diagnosis of AD during life will prove to have non-AD pathology at autopsy [22] and, vice versa, for patients with a clinical presentation suggestive of a non-AD disorder, there is a 39% chance that an autopsy will prove the (co)occurrence of AD pathology [23]. The gold standard for verification of the AD diagnosis is a *postmortem* neuropathological examination. However, correct diagnosis during life is needed, since it allows a more accurate estimation of prognosis and better therapeutic decisions [24,25].

Until now, the pharmaceutical treatment of Alzheimer’s disease was dependent on drugs introduced 20–25 years ago. However, on 7 June 2021, the Food and Drug Administration (FDA) in the USA, approved the anti-amyloid monoclonal antibody aducanumab, as the first disease-modifying treatment for AD in the early clinical stages (MCI, mild dementia) [26]. Aducanumab was approved under the accelerated approval pathway, which requires a long (nine years) post-marketing phase IV study to confirm the drug’s cognitive benefits. Despite the intense discussion, the arguments and debates triggered, all agree that, if such a specific disease-modifying treatment is to be used the diagnosis of AD should be verified with the maximum accuracy as possible.

For in vivo diagnosis, various biomarkers have been studied during the last 25 years, including cerebrospinal fluid (CSF) biomarkers [27]. Among these, three are considered as classical or “core” biomarkers for AD [28]: Aβ_42_, which is decreased in AD and is inversely related to amyloid plaque burden [29]; tau protein phosphorylated to a threonine residue at position 181 (τ_P-181_) which is increased in AD and it is considered as a marker of tangle formation [30]; total tau protein (τ_T_) which is increased in AD and it is a nonspecific marker of neuronal and/or axonal loss [31]. The Aβ_42_/Aβ_40_ ratio may be used instead of Aβ_42_ and seems to perform diagnostically better than the latter [32]. With sensitivities and specificities approaching or exceeding 90%, CSF biomarkers offer added diagnostic value compared to clinically-based diagnosis alone [5] and they have been incorporated in newer diagnostic criteria and guidelines [5,6,7]. A combination of decreased Aβ_42_ with increased τ_P-181_ and τ_T_ is highly specific for the presence of AD, while normal levels of all three biomarkers are highly specific for the absence of AD [33]. Increased levels of the τ_P-181_/Aβ_42_ ratio have also been observed to provide high specificity for the differential diagnosis of AD from other dementias [34]. More recently, the AT(N) classification system has been introduced for diagnostic classification of AD (and possibly other dementia disorders), based on biomarkers [35]. The letter A stands for markers of amyloid pathology, T for markers of tau pathology (tangle formation), and N for markers of neurodegeneration (neuronal/axonal loss). Each letter is followed by either ^+^ or ^−^, representing the positive (abnormal) or negative (normal) result of testing, respectively. The profile (“fingerprint”) of AD is either A^+^T^+^(N)^+^ or A^+^T^+^(N)^−^ [18]. Profiles such as A^+^T^−^(N)^−^ or A^+^T^−^(N)^+^ are compatible with Alzheimer’s *pathological change* (change from normal with the acquisition of amyloid biochemistry/pathology, without or with additional non-AD pathologies), but not Alzheimer’s disease (which requires *both* amyloid plaques and neurofibrillary tangles [1]) [18]. Although the AT(N) system was designed mainly for research purposes, it can be used in clinical practice, even with clinically relevant prognostic value [36] and it may be suitable for in vivo AD verification in patients suitable for aducanumab treatment, especially during the long phase IV trial of aducanumab.

## 2. Patients and Methods

### 2.1. Patients

The four patients presented here were examined at the 2nd Department of Neurology. They had cognitive impairment with an atypical presentation, creating clinical diagnostic uncertainty, with CSF biomarkers resolving the problem by revealing the CSF “neurochemical fingerprint” of AD (otherwise, there were no specific selection criteria).

Initially, history, neurological and complete physical examination were recorded routinely. Secondary causes including thyroid disease, B12 deficiency, neurosyphilis, brain tumor, or subdural hematoma (but not normal pressure hydrocephalus) were excluded. Written informed consent was obtained for all cases. The study had the approval of the Scientific Board and Ethics Committee of “Attikon” Hospital (project identification codes of approval: A13, 7 April 2021 and 157, 16 March 2021 respectively) and was conducted according to the ethical guidelines of the 1964 Declaration of Helsinki.

### 2.2. Neuropsychological Approach

Following history and clinical examination, a battery of neuropsychological tests was performed. Global tests for the assessment of cognition and activities of daily living included the Addenbrooke’s Cognitive Examination-Revised version (ACE-R), the Mini Mental State Examination (MMSE), and the Instrumental Activities of Daily Living (IADL), all of which have been validated in Greece [37,38,39]. Brief bed-side tests for memory (free and cued recall), frontal function, visuospatial skills, and possible depression included the 5-words memory test [40], the Frontal Assessment Battery (FAB) [41], the CLOX (1 and 2) [42], and the short version of the Geriatric Depression Scale (GDS) [43], respectively. Finally, as a tool for the concomitant assessment of cognitive and functional status, the Clinical Dementia Rating (CDR, both sum of boxes and overall score) was used [44].

### 2.3. Neuroimaging

A routine 1.5 or 3T brain magnetic resonance imaging (MRI) scan was the preferred method of neuroimaging, including 3D T1W sequences, suitable for assessing cortical and central atrophy, including medial temporal atrophy, according to a visual scale [45]. The Evans index and callosal angle were also calculated as appropriate [46]. Alternatively, a brain computerized (CT) scan was obtained in cases with MRI contraindication (orthopedic prostheses).

### 2.4. Lumbar Puncture and CSF Biomarker Measurements

A lumbar puncture was performed using a standard, 21–22G, Quincke-type needle, at the L4–L5 interspace, at 9–12 a.m. according to widely accepted recommendations on standardized operative procedures for CSF biomarkers [47]. In brief, CSF was collected in six polypropylene tubes. The first and second tubes (1 mL each) were used for routine CSF cytology and biochemistry, respectively. The third tube (2 mL) was used for oligoclonal bands and IgG index determinations. The following two tubes (5 mL each) were used for biomarker determinations. The last tube (~2 mL) was used for syphilis serology or other tests according to clinical indications. All CSF samples had <500 red blood cells/μL.

The two tubes intended for CSF biomarker analysis were immediately centrifuged (2000× *g* 15 min), aliquoted in polypropylene tubes (1 mL each), and finally stored at −80 °C. Aliquots were thawed only once, just before analysis, which was performed within three months of storage.

Classical CSF biomarkers (Aβ_42_, Aβ_40_, τ_P-181,_ and τ_T_) were measured in a Euroimmun Analyzer I (Euroimmun, Lübeck, Germany), in duplicate, with a double sandwich enzyme-linked immunosorbent assay (ELISA) by commercially available kits (EUROIMMUN Beta-Amyloid (1-42) ELISA, EUROIMMUN Beta-Amyloid (1-40) ELISA, EUROIMMUN pTau(181) ELISA, and EUROIMMUN Total-Tau ELISA, respectively), according to the manufacturer’s instructions and by the use of 4-parameter logistic curves as described elsewhere [48]. All procedures were performed under a stable temperature (21 ± 2 °C) and quality control samples (both in-house and provided by the manufacturer) were used in each run. The inter- and intra-assay coefficients of variation were both <7% for all biomarkers. CSF biomarkers were considered normal according to cut-off values of the Neurochemistry and Biological Markers Unit (Aβ_42_ > 480–500 pg/mL, Aβ_42_/Aβ_40_ > 0.09, τ_P-181_ < 60 pg/mL, τ_T_ < 400 pg/mL).

**Figure 1:**
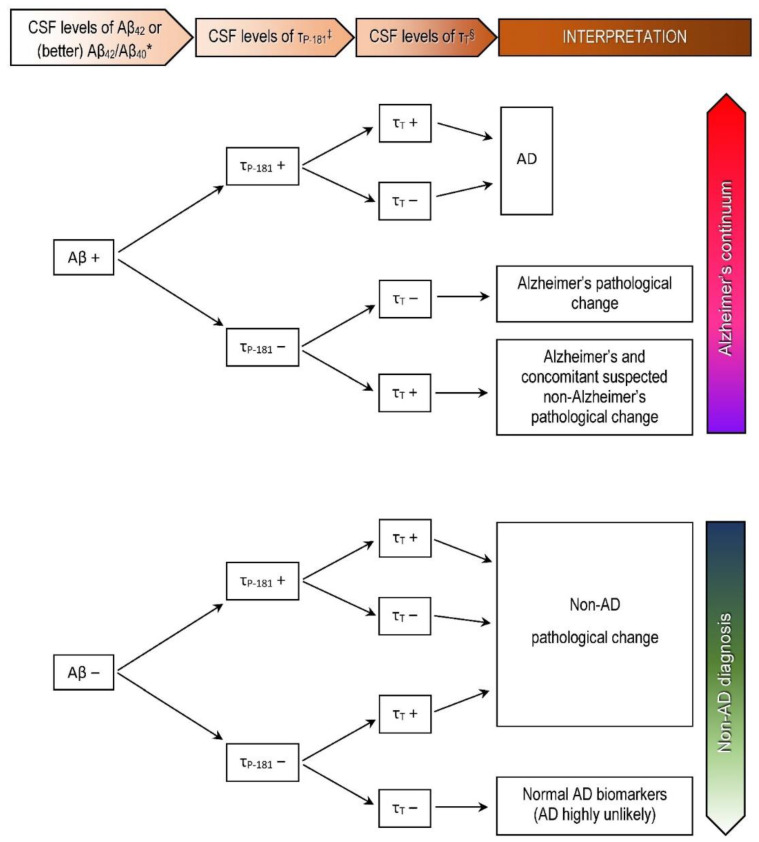
Biomarker levels in the CSF and interpretation of results for clinical purposes in our departments according to the AT(N) classification system, using the classical CSF biomarkers and structural imaging (MRI or CT) [18]. * Abnormal have decreased levels (positive result). ^‡^ Abnormal have increased levels (positive result). ^§^ Abnormal have increased CSF levels or atrophy in structural neuroimaging (positive result). Negative results indicate normal findings. AD: Alzheimer’s disease.

The CSF AD profile (“fingerprint”) was defined as decreased Aβ_42_ or decreased Aβ_42_/Aβ_40_ and increased τ_P-181_, and thus, compatible with the A^+^T^+^(N)^+^ or A^+^T^+^(N)^−^ profiles of the AT(N) classification system [18], according to Figure 1.

## 3. Results

The demographic, clinical, neuropsychological, and CSF neurochemical data of the four patients are summarized in Table 1.

### 3.1. Patient 1

**Figure 2:**
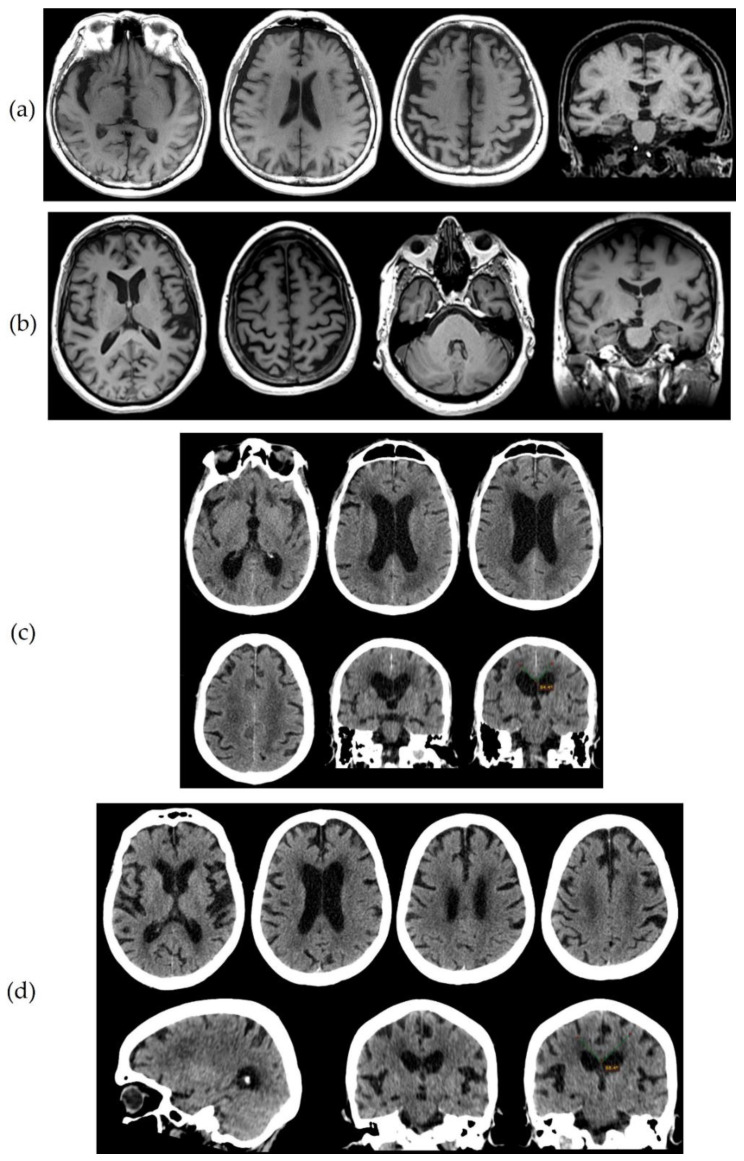
(**a**) T1 Magnetic Resonance Imaging (MRI) sequences of patient 1. Frontal (mainly), frontoparietal, perisylvian, and left hippocampal (grade 3) atrophy is observed. (**b**) T1 MRI sequences of patient 2. Atrophy in the left posterior perisylvian and parietal area is observed with preservation of the hippocampus. (**c**) Computerized tomography (CT) scan of patient 3. Some degree of frontal and parietal atrophy is seen. The white matter shows decreased density consistent with subcortical small vessel disease, in addition to periventricular caps. The parietal convexity is tight, the callosal angle is 84.4° and the Evans index has been calculated to 0.36. (**d**) CT scan of patient 4. Frontal (mainly) and parietal asymmetric atrophy are observed. Although the parietal convexity is not tight, the callosal angle is 88.4° and the Evans index has been calculated to 0.38. Decreased density of the white matter at centrum semiovale is noted, consistent with small vessel disease, with additional periventricular caps.

A seventy-six-year-old female was examined due to four years of “memory problems”. She increasingly had to keep memos and frequently repeated the same questions. According to the results of the neuropsychological testing, she had incipient dementia, with a profile more compatible with a frontal or frontal-subcortical syndrome (decreased attention and concentration and executive function) rather than the typical hippocampal amnestic syndrome (Table 1). Neuroimaging showed frontal–frontoparietal atrophy and asymmetric hippocampal atrophy (Figure 2a). Biomarker assessment showed decreased Aβ_42_ and Aβ_42_/Aβ_40_ ratio and increased both τ_P-181_ and τ_T_, compatible with AD.

### 3.2. Patient 2

This seventy-six-year-old female suffered gradually progressive difficulty in speech for three years. Upon examination, she had a perfect understanding of language, but during spontaneous speech she made many pauses in an effort to “recall” the appropriate word. Upon naming testing, anomic (word-finding) difficulty was obvious, with object knowledge and single-word comprehension completely spared. Phonological errors were frequent and sentence repetition was severely affected. The motor and grammatical aspects of speech were normal. No difficulty in other cognitive domains was reported and decreased scores in neuropsychological testing were attributed mainly to the language (aphasic) disorder. She had no other significant difficulty in activities of daily living except in communication due to the aphasic disorder, which was compatible with Primary Progressive Aphasia (PPA) of the logopenic-type [49]. Atrophy was predominant in the left perisylvian and parietal areas (Figure 2b). Biomarker analysis revealed normal Aβ_42_ with reduced Aβ_42_/Aβ_40_ ratio, together with increased τ_P-181_ and τ_T_, compatible with AD.

### 3.3. Patient 3

An eighty-one-year-old male developed a gradually progressive cognitive decline during the last four years. He had apathy, social withdrawal difficulty in performing complex tasks, mental “slowness”, and reduced attention. The previous year, progressive gait difficulty was noticed, with slow and short steps, sometimes a “magnetic” gait, and occasional falls with one fracture. The previous month, urinary urgency and sometimes incontinence was added into the clinical picture. Neuropsychological testing revealed moderate-stage dementia showing a mixed profile, including significant frontal, amnestic, and visuoconstructive components. Neuroimaging revealed an increased Evans index, acute callosal angle, tight convexity and periventricular caps, suggestive of normal pressure hydrocephalus [46], but cerebral small vessel disease was also evident (Figure 2c). Consistently with the suspicion of normal pressure hydrocephalus, a spinal taping test (removal of 40 mL of CSF) resulted in a significant improvement of gait and cognition. However, CSF biomarkers analysis revealed decreased Aβ_42_ and increased τ_P-181_ and τ_T_, compatible with the additional presence of AD.

### 3.4. Patient 4

This eighty-three-year-old female developed gradually progressive gait difficulty with slow and short steps, postural instability, and frequent falls during the last three years and was unresponsive to L-dopa treatment. In addition, apathy, mental “slowness” and reduced attention were reported. In the previous year, urinary incontinence was noted. Upon clinical examination, she was practically bed-ridden, with asymmetric parkinsonism, including limb bradykinesia and rigidity more evident in the left limbs, while pyramidal signs were additionally present, more evident in the left limbs. Frequent myoclonic jerks were observed in the upper limbs, especially the left. Cortical sensory loss and sensory neglect were present in the right limbs. Primitive reflexes (especially grasping) were also present. Neuropsychological testing revealed moderate-stage dementia showing a mixed profile, including significant frontal, amnestic and visuoconstructive components, while significant upper limb apraxia was present. The patient met clinical criteria for corticobasal syndrome [50]. Despite some degree of asymmetrical atrophy, neuroimaging revealed an increased Evans index, acute callosal angle, and periventricular caps, suggestive of normal pressure hydrocephalus [46], while some degree of cerebral small vessel disease was also evident (Figure 2d). The spinal taping test (removal of 40 mL of CSF) resulted in a significant improvement of cognition, but there was no change in gait. Analysis of CSF biomarkers showed reduced Aβ_42_/Aβ_40_ ratio, together with increased τ_P-181_ and τ_T_, compatible with the presence of AD.

## 4. Discussion

In the present study, we present four cognitively impaired patients with clinical presentations creating diagnostic uncertainty. The first patient was at the transition from MCI to mild dementia and, while she complained of memory problems, the total delayed recall (including memory cues) was normal, which is considered not compatible with the hippocampal amnestic disorder (typically expected in AD), but more compatible with a frontal–subcortical-type of memory decline. Despite a senile onset of disease and a presumably higher probability for AD, this is estimated to be no more than ~70% in such cases with early-stage disease and non-typical presentation [21,22], with other pathologies entering in the differential diagnosis. In the second patient, the clinical profile was compatible with PPA of the logopenic-type, which is due to AD in approximately 50–80% of patients [10,51]. However, it should be not considered synonymous with AD [49], since, in ~25%, it is caused by one of the frontotemporal pathologies [51].

Thus, in both patients 1 and 2, there was still a significant chance (at the level of 25–30%) that a non-AD pathology may be the cause of the cognitive decline. Since both patients had MMSE > 20, making them eligible for aducanumab treatment, it is necessary to increase the diagnostic certainty from 70–75% to as high as possible, in order to initiate such a specific, expensive, and with potentially serious complications, treatment. In both patients, the CSF biomarker results, according to the AT(N) classification system [18], were compatible with the presence of AD.

In patients 3 and 4, the case was quite different since they were mixed cases of dementia. Patient 3 had typical clinical and imaging characteristics of normal pressure hydrocephalus and the positive taping test was consistent with this notion. Normal-pressure hydrocephalus may occur alone, but in three-quarters of cases, AD and/or cerebrovascular disease (usual of the small vessel-type) may be additionally present [52]. In the additional presence of AD, a shunting operation may offer some degree of gait improvement, which may positively affect the quality of life [53]; however, cognitive improvement may be modest [53] and the overall improvement is traditionally thought to be moderate at best and short-lived [54]. Thus, the possible co-occurrence of AD should be known prior to the selection of optimal treatment (or treatment combinations). In patient 3, the whole picture was compatible with NPH and concomitant small vessel disease, both of which may contribute to the clinical picture. However, CSF biomarkers revealed a third significant component in this patient’s dementia, that of AD.

Patient 4 was the most intriguing. She had a mixed movement and cognitive disorder, with a clinical picture typical of corticobasal *syndrome*, while neuroimaging revealed a normal pressure hydrocephalus-like picture and some degree of small vessel disease. A taping test resulted in the improvement of cognition only, but not of gait, probably because the corticobasal component of the motor disability was already severe enough to oppose any improvement. The corticobasal *syndrome* is not a disease, but a clinical picture that can be due to many neurodegenerative diseases, the most common being corticobasal *degeneration* which belongs to the 4-repeat tauopathies [50]. However, it can be caused by AD, Lewy body pathology, progressive supranuclear palsy, and even Creutzfeldt–Jakob disease [12], with AD accounting for a significant percentage of cases with corticobasal *syndrome* [55]. CSF biomarker analysis in patient 4 revealed that AD was indeed the underlying cause. Normal-pressure hydrocephalus was probably present as well (hence the cognitive improvement following the taping test), however, it was superimposed on AD.

Classical CSF biomarkers are useful in identifying the AD biochemical fingerprint in typical and atypical AD cases [27,28]. Their diagnostic performance has been validated in autopsy-proven cases [56]. They have been proven useful in cases with primary progressive aphasia [51], corticobasal syndrome [57], and cases of AD mixed with Lewy body pathology [58] or cerebrovascular disease [14,59]. They can identify the concomitant presence of AD in cases with normal pressure hydrocephalus [60,61], and possibly predict a worse neurosurgical prognosis [62], although recent data suggest that they may predict the opposite [16].

When incorporated in the AT(N) classification system, CSF biomarkers may be used effectively not only in research but also in clinical practice [36,63]. It should be noted that in patients 2 and 4, CSF levels of Aβ_42_ were normal. However, the Aβ_42_/Aβ_40_ ratio was abnormally reduced in both, allowing the diagnosis of AD. Despite some concerns about the interchangeability between Aβ_42_ and the Aβ_42_/Aβ_40_ ratio in the AT(N) system [64], the ratio shows better diagnostic accuracy compared to Aβ_42_ alone [32,65], correlates better with amyloid imaging by positron emission tomography [32], and its better diagnostic performance has been confirmed in pathologically proven cases [32].

There are some limitations in classical CSF biomarker determination. Preanalytical factors, including CSF sampling and storage, may affect test results and internationally accepted guidelines have been formulated for this reason [47]. International quality control programs and projects have been organized, in order to identify and control for confounding factors, improve the methodologies used, optimize analytical performance, and harmonize the levels of biomarkers [66,67,68]. However, there is still a significant intra- and inter-laboratory variability [67,69] and each laboratory should have its own cut-off values [28]. Discordant biomarker results have been observed in different reference laboratories, especially for Aβ_42_ [70]. Diagnostically gray zones also exist and, when added to the possible measurement error, they may lead to a variability of ±25% [70]. Normal levels of all three CSF classical biomarkers may be observed in normal aging, but also in psychiatric disorders which may present with cognitive complaints, sometimes entering in the differential diagnosis of frontotemporal dementia. Furthermore, the classical CSF biomarkers cannot identify additional neurodegenerative pathologies, which are not rare in older patients with AD [71]. Finally, determination of CSF biomarkers requires a lumbar puncture which is a cause of concern and anxiety in many patients and caregivers, and it cannot be easily repeated for frequent follow-up.

Other molecules are under intense investigation in an effort to optimize the differential diagnostic value of the classic biomarkers and identify possible additional neurodegenerative pathologies. They include markers of neuroinflammation such as the triggering receptor expressed on myeloid cells 2 (TREM2), progranulin, and chitinase-3-like protein-1 (YKL-40), markers of synaptic dysfunction such as neurogranin, and markers of neuronal injury such as neurofilament light (NfL) and visinin-like protein 1 (VILIP-1), while miRNAs could also be helpful [72,73,74,75,76,77]. Oligomeric forms of Aβ_42_ [78], α-synuclein [79], and TAR DNA-Binding Protein 43 (TDP43) [80] are emerging biomarkers, but work must still be carried out to achieve adequate diagnostic performance. Especially for α-synuclein, which has been traditionally considered as a marker of synuclein pathology, results are conflicting [79], partially due to the effect of preanalytical and analytical factors, including differences in a-synuclein species detected by different methods [81]. Recent evidence suggests that α-, and also β- and γ-synuclein, may be effective markers of AD rather than synucleinopathy [82]. Both α- and β-synuclein may be early markers of AD, even in non-demented elder subjects [83,84], while the ratio of total tau/α-synuclein may serve as a marker of tau phosphorylation, even allowing patients with the A^−^T^+^(N^+^) profile to re-enter the AD diagnostic group [85]. Blood-based classical [86,87] and exosomal [88] biomarkers may prove helpful, especially for frequent monitoring of the biochemical effects of anti-amyloid antibodies. The AT(N) system is flexible and may expand to an ATX(N) form, incorporating such new or evolving biomarkers of AD-related or additional non-AD pathologies [89].

## 5. Conclusions

Biomarkers are not stand-alone tools and should always be interpreted along with clinical, neuropsychological, and imaging data. Keeping this in mind, analysis of classical CSF biomarkers, especially when incorporated in a classification/diagnostic system such as the AT(N), may offer a significant diagnostic tool [90,91], with both added [92] and prognostic [36] value, allowing the correct identification of AD during life, especially in cases with atypical or mixed presentations [93]. This is always important for correct therapeutic decisions, and it is of paramount importance currently, due to the recent approval of aducanumab as a disease-modifying treatment. Whether atypical cases are going to have the same benefit (from classical or newer treatments) as the typical ones, remains to be elucidated.

## Figures and Tables

**Table 1:** Demographic, clinical, and neurochemical data of the four patients.

	Patient 1	Patient 2	Patient 3	Patient 4
Gender	Female	Female	Male	Female
Age (years)	76	76	81	83
Education (years)	6	12	12	12
Disease duration (years)	4	3	4	3
ACE-R [37]	77/100	51/100	49/100	44/100
MMSE [38]	29/30	23/30	15/30	14/30
IADL [39]	7/8	8/8	3/8	2/8
5-words delayed recall [40]	2 + 3/5	0 + 0/5	0 + 2/5	1 + 1/5
FAB [41]	9/18	10/18	5/18	3/18
CLOX1 [42]	9/15	12/15	0/15	4/15
CLOX2 [42]	10/15	12/15	0/15	6/15
GDS [43]	5/15	4/15	3/15	2/15
CDR sum of boxes [44]	1	0	10	12
CDR overall [44]	0.5	0	2	2
Clinical diagnosis	Incipient dementia (frontal-like?)	PPA logopenic	NPH; VCI	CBS-like; VCI; NPH (?)
Aβ_42_ (pg/mL) (normal > 500)	492.8 ↓	864.5	262.1 ↓	627.9
Aβ_40_ (pg/mL)	13938	12185	NA	11648
Aβ_42_/Aβ_40_ (normal > 0.09)	0.035 ↓	0.071 ↓	NA	0.054 ↓
τ_P-181_ (pg/mL) (normal < 60)	161.6 ↑	110.1 ↑	62.3 ↑	82.6 ↑
τ_T_ (pg/mL) (normal < 400)	557.7 ↑	490.5 ↑	420.1 ↑	427.1 ↑
AT(N) profile [18]	A^+^T^+^(N)^+^	A^+^T^+^(N)^+^	A^+^T^+^(N)^+^	A^+^T^+^(N)^+^
Final diagnosis	AD	AD	NPH + VCI + AD	AD mixed

ACE-R: Addenbrooke’s Cognitive Examination-Revised, MMSE: Mini Mental State Examination, IADL: Instrumental Activities of Daily Living, FAB: Frontal Assessment Battery, GDS: Geriatric Depression Scale, CDR: Clinical Dementia Rating, PPA: Primary Progressive Aphasia, NPH: Normal Pressure Hydrocephalus, CBS: Corticobasal Syndrome, VCI: Vascular Cognitive Impairment, NA: not available. ↓ Decreased levels, ↑ increased levels, ? diagnostic uncertainty remains.

## Data Availability

The data presented in this study are available upon request from the corresponding author. The data are not publicly available due to privacy restrictions.
